# Legal protection of the rights of clinical trial subjects in China

**DOI:** 10.7555/JBR.32.20170073

**Published:** 2017-10-30

**Authors:** Yuanpeng Ren, Xinrui Jin, Shan Jiang, Baisheng Jiang

**Affiliations:** School of Humanities and Social Sciences, Nanjing Medical University, Nanjing, Jiangsu 211166, China.; School of Humanities and Social Sciences, Nanjing Medical University, Nanjing, Jiangsu 211166, China.; School of Humanities and Social Sciences, Nanjing Medical University, Nanjing, Jiangsu 211166, China.; School of Humanities and Social Sciences, Nanjing Medical University, Nanjing, Jiangsu 211166, China.

Subjects in clinical trials, either patients with the target disease or healthy volunteers, inevitably run a risk of injury or even death. To protect human subjects’ rights to life and health, *the Declaration of Helsinki* has been developed as “a statement of ethical principles for medical research involving human subjects”^[1]^. Though widely regarded as a milestone in human research ethics, it is not a law or regulation, and is unable to effectively protect human subjects’ rights. In this context, China beefs up its legal protection of clinical trial subjects.

## Legal regulations in China

Legal protection of clinical trial subjects is stated in national laws of China. Article 26 in *Law of the People’s Republic of China on Medical Practitioners* (implemented on January 5, 1999) provides that physicians should obtain the approval of the hospitals and the consent of the patients themselves or their family members for experimental treatments. Article 29 in *Drug Administration Law of the People's Republic of China *(implemented on January 12, 2001) stipulates that the dossier on a new drug research and development including the manufacturing process, quality specifications, results of pharmacological and toxicological study, and the related data and the samples, in accordance with the regulations of the drug regulatory department under the State Council, should be truthfully submitted to the said authority for approval, before clinical trial is conducted. Article 29 in *Law of the People’s Republic of China on Progress of Science and Technology* (implemented on January 7, 2008) provides that the state should prohibit scientific research and technological development which undermine national security, harm public interests, endanger human health or violate moral principles and ethics.

In China, no specific laws on protection of human subjects have been legislated. Enforcement agencies at all levels have laid down general or specific rules and regulations. It can be said that a roughly complete legal system dominated by administrative rules and normative legal documents has been established for the protection of clinical trial subjects in China. Legal regulations on the protection of human subjects’ rights are listed in chronological order in ***Table 1***. Yet, some of the rules or documents have such lower legal authority and they cannot exercise legal sanctions, like administrative penalty and criminal responsibility, on illegal activities, which in turn weakens the effects of these regulations^[2]^.

**Tab.1:** Legal regulations on the protection of human subjects’ rights

Implementation date	Name	Enactment Organ	Effectiveness level	Overview
10/06/1998	*Interim Measures for the Administration of Human Genetic Resources*	Ministry of Science and Technology, and Ministry of Health, The People’s Republic of China	Administrative rules	To protect and utilize human genetic resources in China. Human genetic resources projects shall follow the principles of mutual benefits, credit and trust joint participation and share of achievements. Any staff member of the administrative department or expert engaging in the examination shall have the duty to keep technological secret for the applicants.
01/09/2003 Revised	*Good Clinical Practice for Pharmaceutical Products* (*GCP*)	China Food and Drug Administration	Administrative rules	To protect the interests and safety of human subjects in clinical trials of new drugs. Ethics committees and informed consent forms are the principal means of ensuring the interests of human subjects.
08/09/2010	*Management Regulations for Ethical Review of Traditional Chinese Medicine Clinical Research*	China State Administration of Traditional Chinese Medicine	Administrative rules	For the ethical review of clinical trials in traditional Chinese Medicine.
02/11/2010	*Guidelines for Ethical Review of Drug Clinical Trials*	China Food and Drug Administration	Normative legal documents	To strengthen the guidance and administration of ethical review of drug clinical trials and standardize the ethical review by ethics committees.
01/03/2015	*Guidelines for International Multi-Center Clinical Trials of Drugs* (trial)	China Food and Drug Administration	Normative legal documents	For the application, implementation and management of international multi-center drug trials conducted in China.
20/07/2015	*Management Methods for Clinical Research on Stem Cells* (trial)	China National Health and Family Planning Commission and China Food and Drug Administration	Normative legal documents	For the management of clinical research on stem cells, aiming to protect human subjects and reduce risks.
01/06/2016	*Good Clinical Practice for Medical Devices*	China Food and Drug Administration and China National Health and Family Planning Commission	Administrative rules	To protect subjects’ rights and safety in clinical trials of medical devices
01/12/2016 Revised	*Ethical Review of Biomedical Research involving Human Beings*	China National Health and Family Planning Commission	Administrative rules	For the ethical review of biomedical research involving human subjects.

All the related regulations in China attach more importance to the subjects’ rights than progress of medical science. The ultimate goal of medical research is to improve humans’ health and quality of life. Article 8 in *Good Clinical Practice for Pharmaceutical Products* stipulates that in the course of drug clinical trials, the rights and interests of the subjects must be adequately safeguarded and the scientificity and reliability of the trial should be ensured and the rights, interests, safety and health of subjects must be higher than the consideration of scientific and social interests.

China has also clarified the validity of international and domestic regulations on the protection of human subjects’ rights. First, foreign organizations and individuals carrying out clinical trials in China must comply with the international and Chinese regulations at the same time. For instance, Article 26 in *Ethical Review of Biomedical Research involving Human Beings* provides that any overseas institution or individual that conducts medical research involving human subjects must submit the research programs for review and approval to Chinese ethical review committee as well as that of their own country or region. Second, the validity of international regulations is superior to that of Chinese regulations in some areas, with the exception of the provisions on which China has announced reservation. For example, Article 4 in *Good Clinical Practice for Pharmaceutical Products* stipulates that all research involving human subjects must conform to the *Declaration of Helsinki of World Medical Association* i.e. fairness, respect of human integrity, maximize the benefits and minimize any harm to the human subject. Third, the laws and regulations across different legal systems within China adhere to the principle of mutual recognition and co-management. For example, *Accreditation of Hong Kong Drug Clinical Trial Institutions and Recognition of Clinical Trial Data* was approved and signed by the China Food and Drug Administration and the Department of Health, the Hong Kong Special Administrative Region after joint review.

## Rights of human subjects 

To protect human subjects’ rights, the first thing is to define their rights, which include the right to life and heath, right to informed consent, right to privacy, right to treatment and right to compensation for damage .

Right to life and health, a fundamental human right confirmed by *Constitution of the People’s Republic of China* and *Civil Law of the People’s Republic of China*, is the foundation of all other human rights and the legislation on clinical trials. In Chinese laws, right to life and health encompasses right to life, right to body and right to health.

Right to informed consent implies that subjects must voluntarily participate in a clinical trial, and be given sufficient time to consider whether to take part in the trial. When a subject lacks legal capacity, his or her legal representative should be informed. Informed consent should use understandable language and words to inform the subjects of the purpose, process and deadline, inspection operation, expected benefits, risks and so on. Subjects’ right to agree or disagree should be respected and safeguarded. Performing the informed consent procedures shall strictly avoid deception, luring, intimidation and other illegal deeds.

Subjects’ right to privacy must be protected according to laws. The storage, usage and security of privacy information should be informed to the subjects, private data should not be exposed to irrelevant third party or media. Revealing subjects’ privacy will infringe their dignity and interests.

When adverse events occur, subjects should have the right to receive timely and appropriate treatment. The sponsor and researcher must guarantee this right, which is of great significance to protect their life and health. If personal injury or even death occurs in clinical trials, subjects or their family members or close relatives have the right to ask for economic compensation. In addition, subjects also have the right to claim compensation under the following circumstances: (1) informed consent is invalid; (2) drugs or equipments are replaced without the agreement of the ethics committee; (3) damage to subjects is due to fault in clinical trials; (4) abnormal damage to subjects occurs unexpectedly after the trials^[3]^.

Above all, the content and scope of the subjects’ rights are comprehensive, which can protect them and strike a balance between human rights’ protection and medical science promotion. Meanwhile, the legal right system is open, so new rights needing legal protection can come forth along with the progress and enrichment of social life.

## Legal regulation enforcement 

As China has not yet enacted independent legislation for the legal rights of subjects, it is generally believed that the ethical review censorship can safeguard clinical subjects’ rights. The ethical committee ensures the subjects’ safety and interests from the perspective of ethics^[4]^. Whether the ethics committee works normatively or not directly affects the scientificity, authenticity, accuracy and reliability of clinical trials, so ethical review must be effectively supervised. Yet, supervision is not satisfactory.

Many problems exist in ethics committee review, such as loose organization structure, unreasonable personnel composition, little training, incapability, non-standard recruitment of members, weak supervision and management mechanism, and unqualified informed consent system, etc^[5–6]^. Therefore, an independent supervision system should be set up to ensure the efficiency of the ethics committee. It is also necessary to establish ethical review project records for tracking and management^[7]^.

Right to informed consent is critical in safeguarding the realization of right to autonomy. Great importance must be attached to individual autonomy. However, there are still some deficiencies in legislation, notification system and consent system, etc. A good informed consent form is a necessary way to help ethics committee improve the quality of protocol review. Researchers should not only inform the subjects, but also accept their consent. Appropriate language expression is important as well.

Meanwhile, China has not fully established a damage insurance system. Once facing physical damage due to trials, subjects need powerful law guarantee and social support. Since subjects have difficulty in providing evidence, “the principle of no fault liability” can be followed^[8]^. Lawyers involved in medical cases assume that informed consent does not mean that the sponsors and researchers should be exempted. They also insist that sponsors and researchers should bear fault liability for their breach of duty.

The scope of compensation is determined by personal damage criteria based on civil law and regulations. Consideration should also be given to appropriate compensation for subjects’ mental damage. Fairness doctrine of reasonable share should be executed for loss. According to various causes of the loss, we should clarify the responsibility of subjects, sponsors, insurance company or the foundation^[9–10]^.

## Legal protection for special subjects

Special subjects in clinical trials should be treated differently. Female subjects in pregnancy and lactation should be paid special attention. Restrictions should be imposed on minors and patients with mental illness and disabilities. Physicians can carry out experimental treatment on subjects for their benefits who temporarily lose decision-making ability due to illness or accident in an emergency. Prisoners, soldiers and subjects’ subordinate staff are strictly restricted to join clinical trials due to their restricted right of self-determination.

Although special subjects have limited capacity for civil conduct, medical trials on them are still justified. Informed consent of special subjects is different from the general one. It is closely related to subjects’ age and mental condition, as well as their cultural background, physical health and freedom of action. Besides informed consent, there should be rational and strict protective measures for special subjects.

As is prescribed in the law, minors in clinical trials should show their agreement. However, *GCP* for minors to participate in trials is more principled, lacks interoperability and has no detailed regulations meeting ethical review standards^[11]^. In addition, *Mental Health Law of the People’s Republic of China* stipulates that only specialists in mental hospital have the right to diagnose mental disorders. If trials must be carried out, mental hospitals should strengthen the construction of ethics committee, and strictly comply with *GCP* and *Mental Health Law of the People’s Republic of China*.

## Conclusion

Legal protection of human subjects’ rights in China is still not satisfactory. On one hand, it is necessary to perfect the existing general laws and special regulations. On the other hand, it is more appropriate to enact special laws to protect the rights of human subjects. Some scholars have drawn up drafts of human trials law. In general, institutionalized protection of subjects’ rights, explicit scope of rights, improvement of ethical review and informed consent, and determination of remedy approach can further protect subjects’ rights.
